# One Health Analysis of *mcr*-Carrying Plasmids and Emergence of *mcr-10.1* in Three Species of Klebsiella Recovered from Humans in China

**DOI:** 10.1128/spectrum.02306-22

**Published:** 2022-10-26

**Authors:** Melissa Chunjiao Liu, Zijuan Jian, Wenen Liu, Junhua Li, Na Pei

**Affiliations:** a BGI Shenzhen, Shenzhen, China; b Department of Clinical Laboratory, Xiangya Hospital, Central South University, Changsha, Hunan, China; c Laboratory of Genomics and Molecular Biomedicine, Department of Biology, University of Copenhagen, Copenhagen, Denmark; d Shenzhen Key Laboratory of Unknown Pathogen Identification, Shenzhen, China; University of Greifswald

**Keywords:** mobile colistin resistance, *mcr-10*, *Klebsiella pneumoniae*, China, One Health, colistin resistance, *mcr*

## Abstract

The global dissemination of the mobile colistin resistance (*mcr*) gene illustrates how the use of colistin in veterinary medicine can affect human health, exemplifying the concept of One Health. This study screened for the existence of *mcr* variants (from *mcr-1* to *mcr-10*) in a 5-year collection of clinical Klebsiella short-read whole-genome sequencing (WGS) data from a tertiary hospital in China (2013 to 2018) and aimed to identify the mechanisms of *mcr* spread. MICs were measured for the *mcr*-positive isolates, and long-read sequencing was performed to complete the *mcr*-positive genome sequences. Six variants (*mcr-1.1*, *mcr-8.1*, *mcr-8.2*, *mcr-9.1*, *mcr-9.2*, and *mcr-10.1*) were identified in 20 genomes, with plasmids from the IncFII_K_, IncHI2, IncI2, and IncX4 groups. Highly similar plasmids (coverage, >75%; nucleotide identity, >98.5%) isolated from silver gulls, chickens, pigs, wastewater treatment plants, and hospital sewage were identified in GenBank. The MICs of the *mcr-1*- and *mcr-8*-carrying isolates were ≥4 μg/mL; however, the MICs of the *mcr-9*- and *mcr-10*-carrying isolates ranged from 0.5 μg/mL to 1 μg/mL (colistin susceptible). The variants *mcr-1* to *mcr-9* were found only in Klebsiella pneumoniae, while *mcr-10.1* was found in K. pneumoniae, Klebsiella quasipneumoniae subsp. *quasipneumoniae*, and Klebsiella variicola. A pair of inverted repeats (IRs) was identified for *hsdSMR*-IS*Ec36*-*mcr-10.1*-*xerC*; IR-1 (5′-TCAAACGTA) was inside the *att*L site of *xerC*, indicating that *mcr-10.1* was originally integrated by *xerC* and mobilized by IS*Ec36* afterwards. In conclusion, this is the first report of *mcr-10.1* susceptible to colistin in three species of Klebsiella. This study shows the genetic events that happened to *mcr-10.1* in a stepwise manner, with the first step being XerC integration and the second being IS*Ec36* mobilization. Finally, this study also highlights *mcr* transmission between humans and nature.

**IMPORTANCE** Reports of *mcr-1* and *mcr-8* are common in China; however, few studies have reported *mcr-9* and *mcr-10*. One reason is that the newly described variants can be phenotypically colistin susceptible and thus may not be identified. This study identified the *mcr*-positive clinical isolates by investigating WGS data for 2,855 Klebsiella isolates (including K. pneumoniae, K. quasipneumoniae subsp. *quasipneumoniae*, and K. variicola) and found three *mcr-9* and three *mcr-10* cases (MICs, 0.5 μg/mL to 1 μg/mL; colistin susceptible). This study also reveals a pair of perfect 9-bp IRs of IS*Ec36* and the precise *mcr-10.1* integration and insertion events that happened to the IncFII_K_ plasmids. A One Health analysis of highly similar plasmid structures from human and nonhuman sources emphasizes the plasmid transmission and evolution process.

## INTRODUCTION

Colistin (polymyxin E) is extensively used in treating food-producing animals but was banned from human use in the 1970s due to its nephrotoxicity and neurotoxicity (1). However, with the evolution and increase in multidrug-resistant (MDR) bacteria, particularly carbapenem-resistant *Enterobacteriaceae* (CRE), in hospitals around the world, colistin has been reintroduced to clinical medicine. Unfortunately, resistance to colistin subsequently appeared in both human and nonhuman cases, indicating a zoonotic disseminating issue which should be addressed using a One Health perspective (2–4).

Colistin resistance can be caused by either chromosomal mutations or mobile colistin resistance (*mcr*) gene acquisition (5). In *Enterobacteriaceae* strains, *mcr* acquisition is more concerning, as the *mcr* gene can rapidly disseminate resistance via horizontal gene transfer (HGT). The *mcr* gene encodes a phosphoethanolamine transferase that can modify the lipid A component of lipopolysaccharide. The common mobile genetic elements (MGEs) assisting *mcr* mobilization include insertion sequences (ISs; e.g., IS*Apl1*, IS*903B*, IS*Ecl1*, IS*Kpn26*, etc.), recombinases, integrases, and plasmids from the IncI, IncH, IncX, and IncF incompatibility groups (3, 6).

As of 2021, a total of 10 *mcr* homologs have been identified (6). The first to be reported was *mcr-1* from pigs and chickens in China (7). Since then, *mcr-1* has been widely described, especially in Escherichia coli (8, 9). In contrast, the other *mcr* variants have been reported in a limited number of species (10). In Klebsiella, *mcr-8* and *mcr-9* have been reported. The *mcr-8* gene was initially identified in the K. pneumoniae plasmid pKP91 of swine origin (11), and *mcr-9* was detected *in silico* from a clinical colistin-susceptible Salmonella enterica serotype Typhimurium strain isolated in 2010 in the United States (12). The expression of *mcr-9* normally requires exposure to colistin and a *qseBC* system next to *mcr-9* for expression induction (3). Notably, the IncHI2 plasmid is the predominant plasmid type disseminating *mcr-9* (13); this plasmid is widely known for its carriage of multiple antimicrobial-resistant genes (ARGs), including the carbapenemase genes (3). The latest identified *mcr* variant is *mcr-10*, which was originally detected in the clinical Enterobacter roggenkampii strain 090065 in China, showing colistin MIC values ranging from 2 μg/mL (susceptible) to 4 μg/mL (resistant) (6). High-level colistin resistance (MIC, ≥16 μg/mL) of *mcr-10* could be screened out using high concentrations of colistin (4 μg/mL to 8 μg/mL) (2). High mRNA levels of *phoPQ* were observed for the high-level resistance induced by the high colistin pressures. The *mcr-10* variants have been reported in IncF plasmids, including the IncFIA, IncFIB, and IncFII groups, in six genera (Enterobacter, Klebsiella, Escherichia, *Citrobacter*, *Kluyvera*, and *Raoultella*) (6).

In this study, we (i) conducted a genome surveillance study of clinical *mcr* variants in K. pneumoniae over 5 years, (ii) determined the clinical features and phenotypic impacts of the *mcr*-carrying isolates, (iii) characterized the *mcr*-carrying isolates from species level to the *mcr*-surrounding genetic context, and (iv) linked the *mcr*-carrying plasmids from human sources to plasmids from other sources in nature.

## RESULTS

### Overview of the 20 *mcr*-carrying K. pneumoniae genomes.

Of the 2,855 Klebsiella genomes, 20 (0.70%) were positive for *mcr*. The median age of the infected patients was 52 years (ranging from 1 month to 78 years), and 17 (85%) were male (Table 1). Eleven patients were hospitalized, and nine were outpatients. The isolates were isolated from sputum (*n *= 9), blood (*n *= 7), urine (*n *= 2), drainage fluid (*n *= 1), and wound secretions (*n *= 1). Notably, 18 were MDR with phenotypic resistance to more than three antibiotic categories, and 17 were extended-spectrum beta-lactamase (ESBL) producers. Only four were carbapenem resistant and carried *bla*_NDM-5_. Comorbidities included cerebral injury (*n *= 4), malignant spinal tumor (*n *= 1), severe acute pancreatitis (*n *= 1), gastric cancer (*n *= 1), coronary artery disease (*n *= 1), aplastic anemia (*n *= 1), and acute lymphoblastic leukemia (ALL; *n *= 1; died with isolates carrying both *mcr-8.1* and *bla*_NDM-5_).

**TABLE 1:** Clinical information and genomic characterization of the 20 *mcr*-carrying plasmids

Strain	Yr	Age (yr)	Gender^*a*^	Specimen type	Hospitalized	MDR	ESBL	CRE	*mcr* variant	Colistin MIC	Sp.^*b*^	MLST	Inc group^*c*^	Plasmid size (kb)
29-72	2014	49	M	Sputum	+	−	−	−	1.1	4	KpI	ST43	X4	34
15-81	2016	78	M	Sputum	+	+	+	−	1.1	4	KpI	ST1	X4	34
17-6	2016	66	F	Blood	+	+	+	−	1.1	16	KpI	ST268	I2	66
17-66	2016	19	M	Sputum	+	+	+	−	1.1	16	KpI	ST268	I2	66
1-17	2017	38	M	Abdominal drainage	−	+	+	+	1.1	16	KpI	ST5258	FII_K_	66
5-30^*d*^	2017	8	M	Sputum	−	+	+	−	1.1	8	KpI	ST39	HI2	266
19-40	2016	37	M	Sputum	+	+	+	+	8.1	8	KpI	ST685	FII_K_	95
38-73	2016	37	M	Blood	+	+	+	+	8.1	8	KpI	ST685	FII_K_	95
38-72	2016	37	M	Blood	+	+	+	+	8.1	8	KpI	ST685	FII_K_	104
1-36	2017	52	M	Urine	−	+	+	−	8.1	>64	KpI	ST395	FII_K_	110
1-44	2017	70	M	Blood	−	+	+	−	8.1	>64	KpI	ST395	FII_K_	110
4-35	2017	70	M	Sputum	−	+	+	−	8.1	>64	KpI	ST395	FII_K_	110
29-21	2015	63	M	Blood	+	+	−	−	8.2	4	KpI	ST39	FII_K_	86
11-62	2016	58	M	Sputum	−	+	+	−	8.2	4	KpI	ST1	FII_K_	97
36-80^*d*^	2018	68	M	Sputum	−	+	+	−	9.1	1	KpI	ST412	HI2	242
28-33	2015	44	F	Urine	−	+	+	−	9.2	1	KpI	ST3687	HI2	248
28-1-0	2015	66	M	Wound	−	+	+	−	9.2	1	KpI	ST3687	HI2	251
31-62^*d*^	2014	66	M	Blood	+	−	−	−	10.1	1	KpI	ST1087	FII_K_	153
25-52	2015	16	F	Blood	+	+	+	−	10.1	1	KpII-A	ST5281	FII_K_	91
15-76	2016	0.1	M	Sputum	+	+	+	−	10.1	0.5	KpIII	ST5266	FII_K_	128

a Gender: M, male; F, female.

b *Sp*., species complex; KpI, K. pneumoniae; KpII-A, *K*. *quasipneumoniae* subsp*. quasipneumoniae*; KpIII, *K. variicola*.

c Inc group, incompatibility group.

d Completed by hybrid assembly using both short and long reads.

A total of six *mcr* variants were identified in the 20 genomes, including *mcr-1.1* (*n *= 6), *mcr-8.1* (*n *= 6), *mcr-8.2* (*n* = *2*), *mcr-9.1* (*n *= 1), *mcr-9.2* (*n *= 2), and *mcr-10.1* (*n *= 3). All but two *mcr*-positive genomes were K. pneumoniae. The remaining two were Klebsiella quasipneumoniae subsp*. quasipneumoniae* and Klebsiella variicola, both of which carried *mcr-10.1*. MLST revealed that the *mcr*-carrying genomes were from a variety of clones, indicating that the *mcr* dissemination was not due to clonal spread. Both the *mcr-9*- and *mcr-10*-carrying isolates were colistin susceptible (*n *= 6; MIC, 0.5 μg/mL to 1 μg/mL), while the MIC values of the remaining 14 were ≥4 μg/mL. No *qseBC* gene was found in the genomic contigs of the *mcr-*carrying isolates. Chromosomal genes (*phoP/phoQ*, *pmrA/pmrB*, and *mgrB*) known to be associated with colistin susceptibility and *mcr* expression regulation were investigated by amino acid sequences aligned to the reference gene sequences of K. pneumoniae MGH 78578 (GenBank accession number CP000647.1) (see Fig. S1 in the supplemental material). *phoQ* and *pmrAB* sequences in *mcr-10.1*-carrying K. quasipneumoniae subsp. *quasipneumoniae* and K. variicola genomes had more nonsynonymous mutations (*n *= 36) than the sequences in the *mcr-10.1*-carrying K. pneumoniae genome (*n *= 1).

Plasmid typing revealed that the most common *mcr*-carrying plasmid type was IncFII_K_ (*n *= 12), followed by IncHI2 (*n *= 4), IncI2 (*n *= 2), and IncX4 (*n *= 2). Further investigation of the *mcr* genetic contexts was performed, and relatively consistent genetic patterns were found, i.e., IS*Apl1* upstream of *mcr-1.1* and *pap2* (IS*Apl1*-*mcr-1.1*-*pap2*; *n *= 4); IS*903B* downstream of *mcr-8.1* with no flanking transposons upstream (*dgkA*-*beaS*-*copR*-*mcr-8.1*-//-IS*903B*; *n *= 6); IS*Ecl1* upstream of *mcr-8.2* (IS*Ecl1*-*mcr-8.2*-//-IS*Kpn26*; *n *= 2); IS*903B* upstream of *mcr-9.1* and *mcr-9.2* (IS*903B*-*mcr-9*; *n *= 3); and *xerC* upstream of *mcr-10.1* (*xerC*-*mcr-10.1*; *n *= 3) (Fig. S2).

### Genetic characterization of the *mcr*-carrying plasmids.

**FIG 1:**
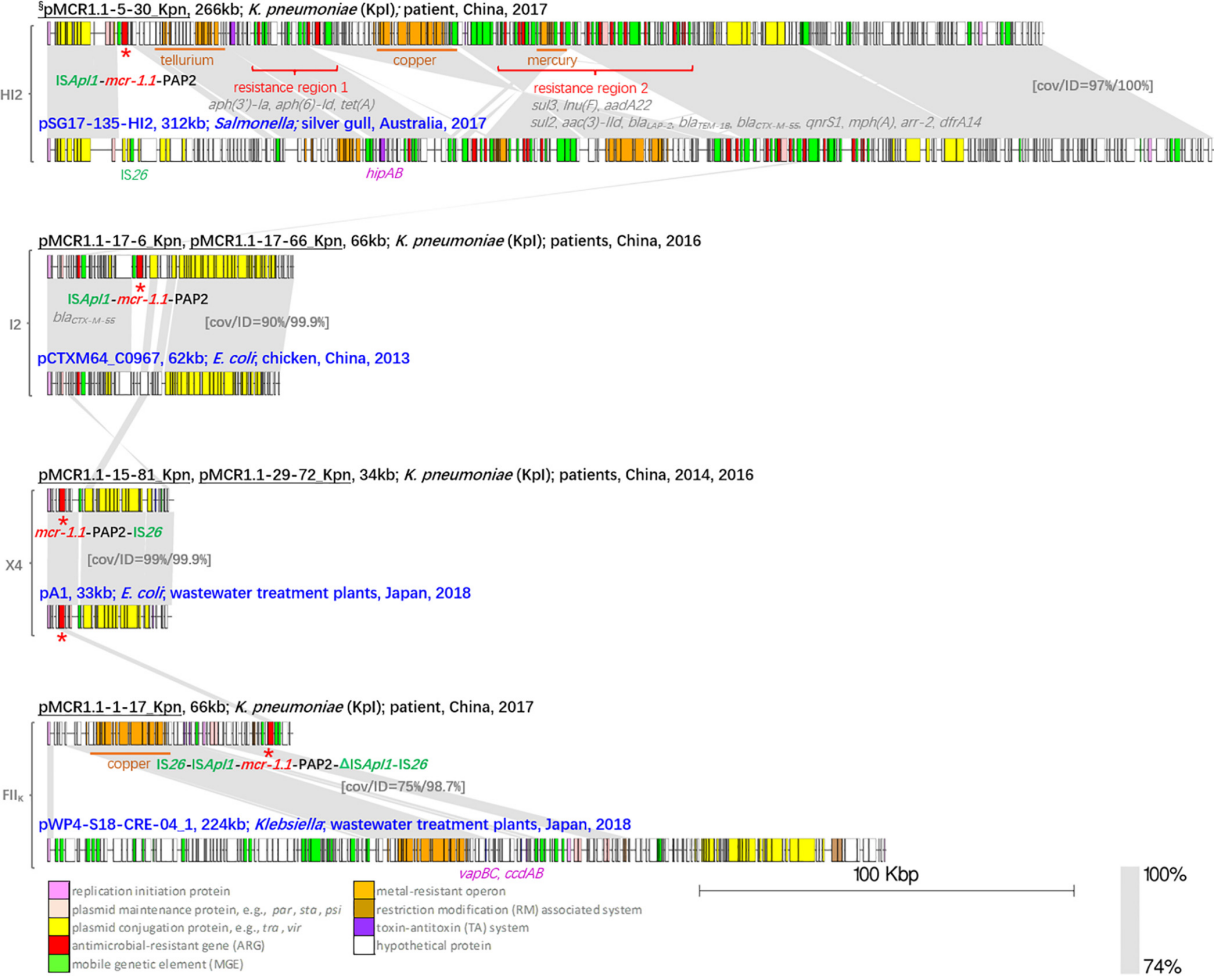
Comparison of plasmids harboring *mcr-1.1* obtained in this study with those reported from nonhuman sources. The complete plasmids are grouped by the incompatibility groups IncHI2, IncI2, IncX4, and IncFII_K_. The plasmid name, length in kilobases, species, and isolation information (source, location, and year) are shown above each sequence. Plasmids from this study are labeled in black, and plasmids from nonhuman sources are labeled in blue. §, plasmid sequences that were completed by hybrid assembly; Δ, truncated insertion sequences. The *mcr* genes are indicated by a red asterisk (*), and the *mcr* genetic context arrays are shown next to the asterisk. Heavy metal resistance operons and resistance regions are labeled in orange and red, respectively. Gray shading connects highly similar sequences. cov, coverage; ID, identity.

Of the six *mcr-1.1*-positive plasmids, one was carried by an IncHI2 plasmid, two by IncI2 plasmids, two by IncX4 plasmids, and one by an IncFII_K_ plasmid. It is interesting to note that pMCR1.1-5-30_Kpn (completed by hybrid assembly using both short and long reads), identified in this study, shared a similar IncHI2 backbone with pSG17-135-HI2 (GenBank accession number CP048776.1), a plasmid isolated from a gull in Australia (Fig. 1). Both pMCR1.1-5-30_Kpn and the *mcr*-free plasmid pSG17-135-HI2 carried resistance region 1 (three ARGs) and resistance region 2 (12 ARGs), conferring resistance to aminoglycosides [*aph(3′)-Ia*, *aph(6)-Id*, *aadA22*, and *aac(3)-IId*], tetracycline [*tet*(A)], sulfonamides (*sul3* and *sul2*), lincomycin [*lnu*(F)], beta-lactams (*bla*_LAP-2_, *bla*_TEM-1B_, and *bla*_CTX-M-55_), quinolone (*qnrS1*), macrolides [*mph*(A)], rifamycin (*arr-2*), and trimethoprim (*dfrA14*), in addition to colistin (*mcr-1.1*). In addition, three heavy metal resistance operons (*terABCDWXY*, *cusABCDRS*-*copABCDEFG*-*pcoERS*, and a truncated *merCDEPTR*) and one toxin-antitoxin (TA) system (*hipAB*) were identified in pMCR1.1-5-30_Kpn. Shorter *mcr-1.1*-carrying plasmids were found in the other three plasmid incompatibility groups. The closest reference of the IncI2 *mcr-1.1*-carrying plasmids was pCTXM64_C0967 (KP091735.1), a *bla*_CTX-M-64_-carrying and *mcr*-free plasmid from a chicken source. An average nucleotide identity (ANI) of 99.9% was found between them. The linear map indicated that the colocation of *mcr-1.1* and *bla*_CTX-M_ evolved from the *bla*_CTX-M_-carrying plasmid. A 99.9% ANI and 99% coverage were found between the *mcr-1.1*-carrying IncX4 plasmids identified in this study and pA1 (LC477138.1), a *mcr-1.1*-carrying plasmid from a Japanese E. coli strain from a wastewater treatment plant. Another plasmid isolated from a wastewater treatment plant in Japan, pWP4-S18-CRE-04_1 (AP022079.1), was a close match of pMCR1.1-1-17_Kpn. The copper resistance operon and the TA systems of *vapBC* and *ccdAB* were shared between them; however, no IncFII_K_ *tra* region was identified in the genomic contigs of pMCR1.1-1-17_Kpn.

**FIG 2:**
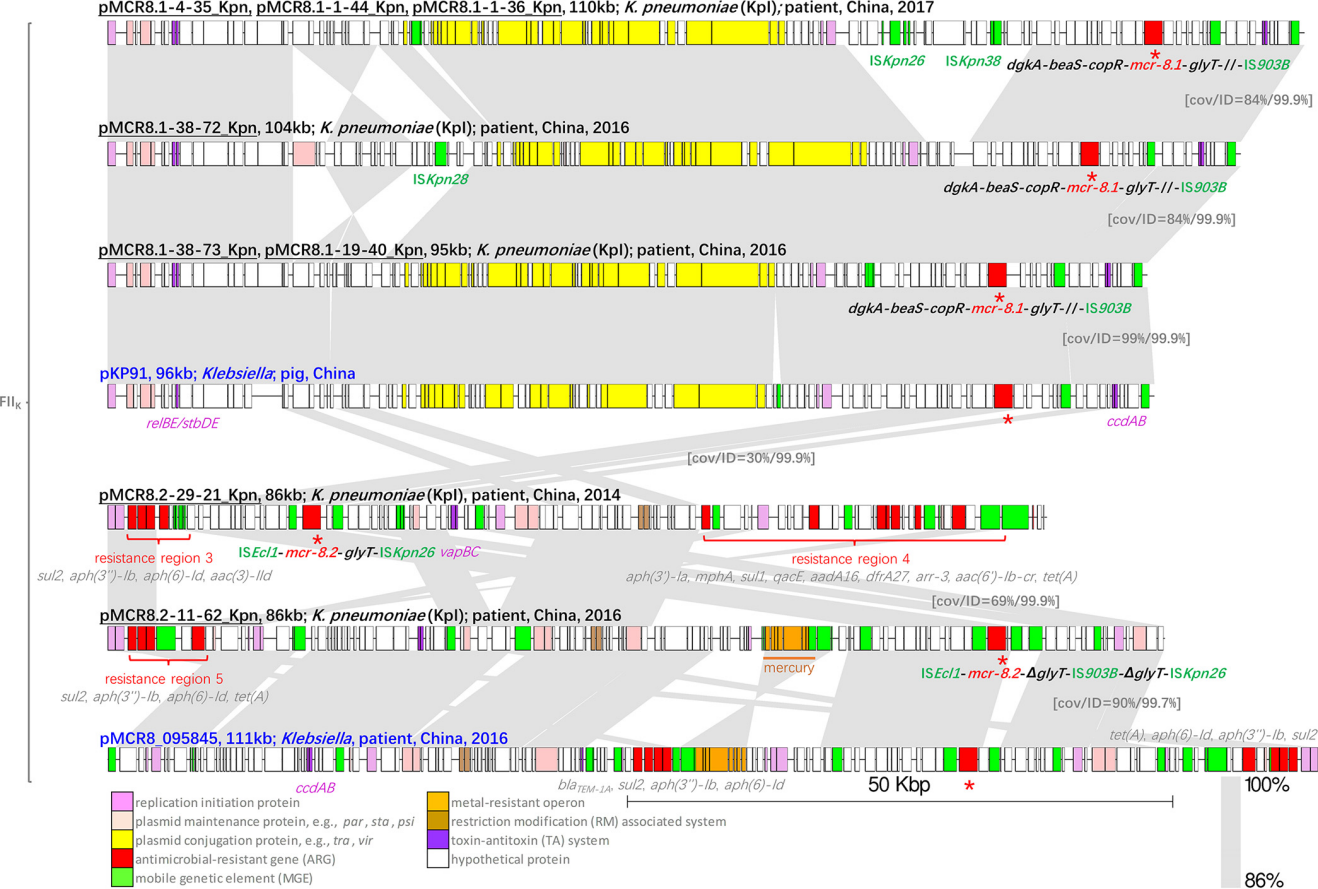
Comparison of IncFII_K_ plasmids harboring *mcr-8* obtained in this study and those reported from nonhuman sources. The plasmid name, length in kilobases, species, and isolation information (source, location, and year) are shown above each sequence. Plasmids from this study are labeled in black, and plasmids from previous studies are labeled in blue. The *mcr* genes are indicated by a red asterisk (*), and the *mcr* genetic context arrays are shown next to the asterisk. Heavy metal resistance operons and resistance regions are labeled in orange and red, respectively. Gray shading connects highly similar sequences. cov, coverage; ID, identity.

All eight *mcr-8*-carrying plasmids were from the IncFII_K_ group. However, the IncFII_K_ plasmid backbone of the *mcr-8.1*-carrying plasmids differed from that of the *mcr-8.2*-carrying plasmids, indicating that the transmission dynamics of the dominant *mcr* variants in this study were diversified (Fig. 2). The *mcr-8.1*-carrying plasmids shared a more conserved backbone with a long *tra* region, while the *mcr-8.2*-carrying plasmids shared a less conserved backbone, showing higher genetic plasticity with multiple resistance regions. The clinical *mcr-8.1*-carrying plasmids in this study showed 99.9% ANI with pKP91 (GenBank accession number MG736312.1), the initial report of *mcr-8.1* in K. pneumoniae. The longest *mcr-8.1*-carrying plasmids (110 kb) were all isolated in 2017 and harbored an additional fragment containing heat shock proteins mobilized by multiple insertion sequences, mainly IS*Kpn26* and IS*Kpn38*, compared to the shorter *mcr-8.1*-carrying plasmids isolated in 2016. Plasmid pMCR8.1-38-72_Kpn possessed an IS*Kpn28* inserted region composed of the plasmid partition protein ParB, the single-stranded DNA binding protein Ssb, the reverse transcriptase protein LtrA, and the SOS inhibitors PsiA and PsiB. By comparison, the *mcr-8.2*-carrying plasmids were less conserved, with multiple regions being inserted, and no close match was found in nonhuman sources. The clinical plasmid pMCR8_095845 (CP031883.1) was similar to pMCR8.2-11-62_Kpn, with resistance region 5 (four ARGs), *ccdAB*, antirestriction systems, and one mercury resistance operon (*merCDEPTR*). In pMCR8.2-29-21_Kpn, resistance region 3 differed from resistance region 5 (four ARGs) by only one gene [*aac(3)-IId* in region 3 versus *tet*(A) in region 5]. Moreover, a larger resistance region 4, constituting nine ARGs, was identified in pMCR8.2-29-21_Kpn.

**FIG 3:**
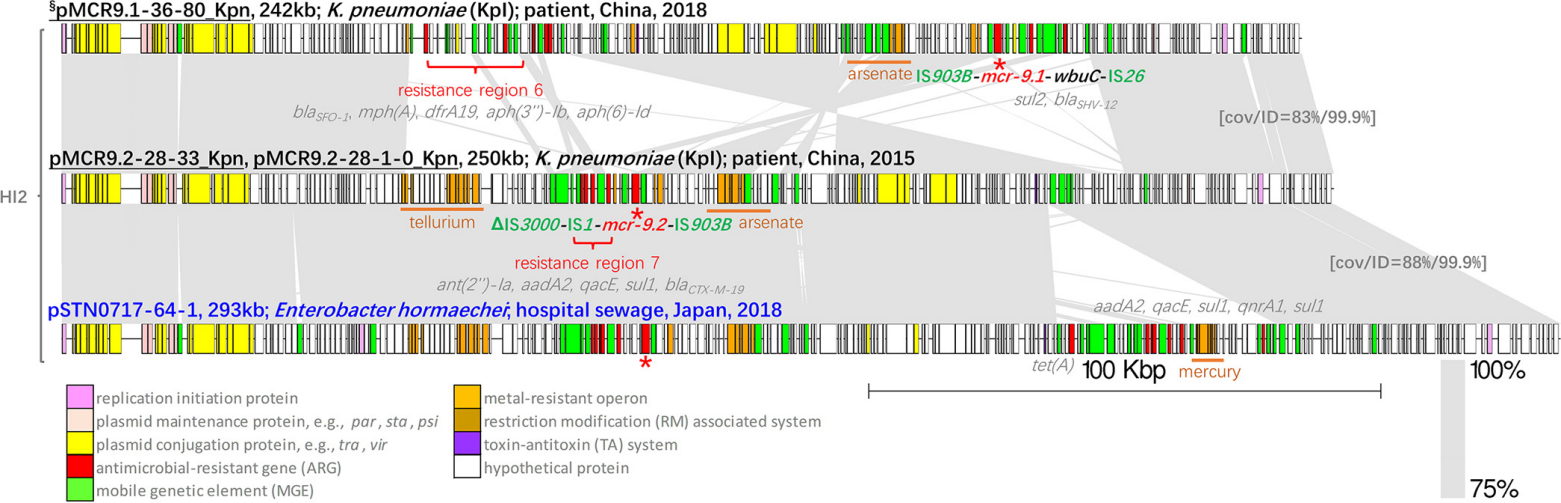
Comparison of IncHI2 plasmids harboring *mcr-9* obtained in this study and a plasmid from a nonhuman source. The plasmid name, length in kilobases, species, and isolation information (source, location, and year) are shown above each sequence. Plasmids from this study are labeled in black, and pSTN0717-64-1 (GenBank accession number AP022511.1) is labeled in blue. §, plasmid sequences that were completed by hybrid assembly. The *mcr* genes are indicated by a red asterisk (*), and the *mcr* genetic context arrays are shown next to the asterisk. Heavy metal resistance operons and resistance regions are labeled in orange and red, respectively. Gray shading connects highly similar sequences. cov, coverage; ID, identity.

Three large IncHI2 plasmids were obtained from the *mcr-9*-carrying genomes. Despite a large fragment reversed between the *mcr-9.1*-carrying pMCR9.1-36-80_Kpn (completed by hybrid assembly) and the *mcr-9.2*-carrying pMCR9.2-28-33_Kpn and pMCR9.2-28-1-0_Kpn, all plasmids shared a conserved IncHI2 plasmid backbone (coverage, >83%; ANI, >99.9%) (Fig. 3). Insertion of resistance regions is the main reason for the large plasmid sizes. pMCR9.1-36-80_Kpn possessed resistance region 6, consisting of five ARGs, and the *mcr-9.2*-carrying plasmids harbored resistance region 7, also composed of five ARGs. Their closest match, pSTN0717-64-1 (GenBank accession number AP022511.1) from hospital sewage in Japan, also contained resistance region 7 and an additional ARG array, ranging from *tet*(A) to *sul1*. No *qseBC* gene was identified in the contigs of the *mcr-9*-carrying genomes.

### Diversified genetic contexts of *mcr-10.1* due to insertions.

Three *mcr-10.1*-carrying IncFII_K_ plasmids were identified in three Klebsiella species. The genetic contexts of *mcr-10.1* were relatively conserved, with an immediately upstream *xerC* and a downstream IS*Ec36* (Fig. S2d). Although the genetic context in pMCR10.1-15-76_Kpv was interrupted by an IS*Kox1*, the flanking sequences downstream of IS*Ec36* remained unchanged.

**FIG 4:**
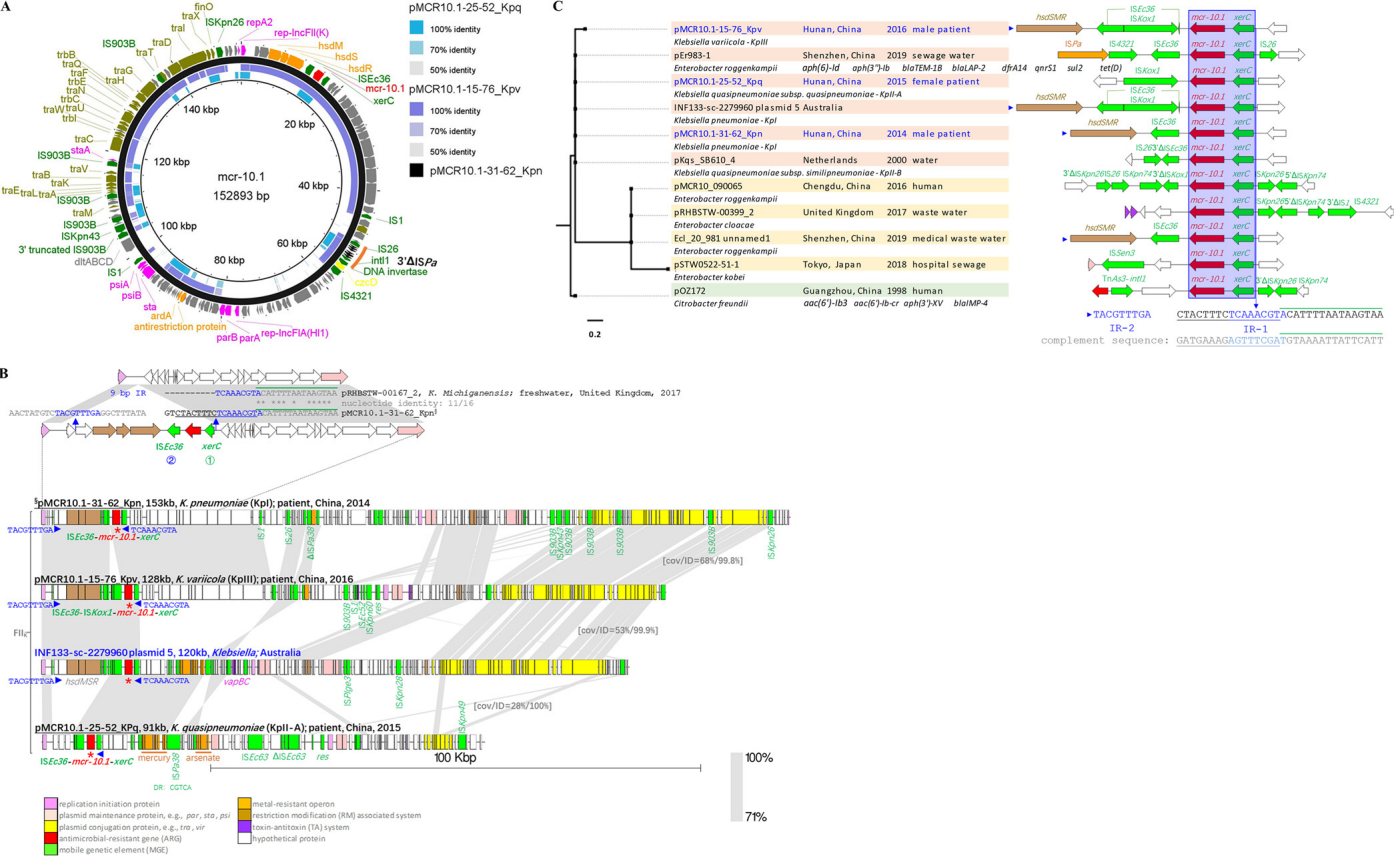
Comparison of the *mcr-10.1*-carrying IncF plasmids. (A) Circular map showing the three *mcr-10.1*-carrying IncFII_K_ plasmids identified in this study. All plasmids were aligned to pMCR10.1-31-62_Kpn (completed by hybrid assembly; 152,983 bp). Functional genes are labeled in different colors. (B) Schematic representation showing the *hsdSMR*-IS*Ec36*-*mcr-10.1*-*xerC* insertion event and comparison of the *mcr-10.1*-carrying IncFII_K_ plasmids. The 9-bp inverted repeats (IRs) are indicated in blue. The two 16-bp parts of the entire 32-bp *xerC att*L sequence are indicated by underlining and a green overline. Identical bases are indicated by asterisks (*). (C) Phylogenetic tree of the *mcr-10.1*-carrying IncF plasmids. The isolation information (source, location, and year), *mcr-10.1* genetic contexts, and additional antimicrobial-resistant genes are shown. The 9-bp IRs are indicated with arrows and highlighted in blue text. The 32-bp *xerC att*L site is indicated by underlining and overlines. The complement sequence of *att*L is also shown. Δ, truncated insertion sequences.

The complete sequence of pMCR10.1-31-62_Kpn was obtained by hybrid assembly. Plasmid pMCR10.1-15-76_Kpv from K. variicola showed 53% coverage with pMCR10.1-31-62_Kpn from K. pneumoniae, while pMCR10.1-25-52_KPq from K. quasipneumoniae subsp. *quasipneumoniae* only shared 19% (Fig. 4A). All three plasmids contained the main genetic elements of the replication-associated genes of IncFII_K_, the IS*Ec36*-*mcr-10.1-xerC* genetic context, a 3′ truncated IS*Pa38* from the Tn*3* family, and parts of the IncFII_K_ *tra* region. The *mcr-10.1*-carrying plasmids obtained in 2014 and 2016 during this study showed a relatively high diversity compared to the other *mcr*-carrying plasmids in this study.

To fully understand the integration and insertion events of *mcr-10.1* in our IS*Ec36*-*mcr-10.1-xerC*-carrying plasmids, a BLAST search for highly similar sequences was performed, and the *mcr-*free IncFII_K_ plasmid pRHBSTW-00167_2 (GenBank accession number CP058119.1) was noted. By carefully comparing pRHBSTW-00167_2 and pMCR10.1-31-62_Kpn, a pair of 9-bp perfect IRs were identified (Fig. 4B). IR-1 (5′-TCAAACGTA) was 95 bp upstream of *xerC* and located inside the 32-bp left attachment site (*att*L; 5′-CTACTTTCTCAAACGTACATTTTAATAAGTAA), and IR-2 (5′-TACGTTTGA) was located downstream of *mcr-10.1* and a type I restriction-modification system encoded by *hsdSMR*. Two 16-bp *att*L parts contained 11 identical nucleotides. No *att*R was identified in our three *mcr-10.1*-carrying plasmids. The presence of perfect IRs and the absence of *xerC att*R indicated that the IS*Ec36* insertion event happened after *xerC* integration and destroyed the complete *xerC att*R site. The plasmid linear map further revealed that the *mcr-10.1*-carrying plasmids were diversified by a variety of ISs. However, no additional ARG was found in the *mcr-10.1*-carrying IncFII_K_ plasmids. The mercury-resistant and arsenate-resistant operons were found in the shortest *mcr-10.1*-carrying plasmid, pMCR10.1-25-52_KPq from K. quasipneumoniae subsp. *quasipneumoniae*. In addition, a complete IS*Pa38* (also called Tn*Pa38*) was identified in pMCR10.1-25-52_KPq, with a pair of 5-bp direct repeats (DRs) of 5′-CGTCA. The closest sequence from a public database was the IncFII_K_ plasmid 5 in the K. pneumoniae genome of INF133-sc-2279960 (LR890193.1) from Australia. This plasmid also contained the *hsdSMR*-IS*Ec36*-*mcr-10.1-xerC* genetic context, with the 9-bp IRs present on both sides.

In addition, the phylogenetic structure of *mcr-10.1*-carrying IncF plasmids and additional *mcr-10.1* genetic contexts were inspected to understand *mcr-10.1* evolution. The maximum likelihood trees divided 11 highly related *mcr-10.1*-carrying plasmids into two main groups (Fig. 4C). The *mcr-10.1*-carrying IncFII_K_ plasmids from this study were grouped into one with homologous IncFII plasmids from water. The *mcr-10.1*-carrying IncFIA and IncFIB plasmids formed another group. Noticeably, *xerC* was the only conserved MGE adjacent to *mcr-10.1*. The downstream of *mcr-10.1* was divergent. IR-1 located exactly in *xerC att*L was present in all *mcr-10.1*-carrying plasmids, while IR-2 only existed in the *hsdSMR*-IS*Ec36*-*mcr-10.1*-*xerC*-carrying sequences.

## DISCUSSION

This study investigated the genetic relatedness of *mcr*-carrying plasmids in Klebsiella isolates recovered from humans in China with publicly available plasmids from nonhuman sources using a One Health perspective and aimed to establish potential links to understand the ways in which the plasmids evolved and were disseminated between animals, humans, plants, and the environment. This study also characterized the prevalence, clinical features, and phenotypic characteristics of the clinical *mcr*-positive Klebsiella genomes. It is the first worldwide report of *mcr-10.1* susceptible to colistin in clinically uncommon species of *K. quasipneumoniae* subsp*. quasipneumoniae* and *K. variicola*. The *mcr-10.1* was found embedded in a genetic structure (*hsdSMR*-IS*Ec36*-*mcr-10.1*-*xerC*) with a pair of IRs.

This study demonstrates that approximately 0.70% of clinical Klebsiella genomes were *mcr* positive in a 5-year period before the implementation of the colistin withdrawal policy in China in 2018 (14). The overall *mcr*-positive rate is comparable with other reports (4, 7). The *mcr-8* homolog is predominant in our collection, with *mcr-1* being the second most common. Unlike the situation in Australia (3), cocarriage of the carbapenemase gene and *mcr* was rare in this study, occurring in only one isolate; however, the infected patient was dead in this study (15).

The identification of *mcr* in both human and nonhuman sources, along with the fact that colistin is heavily used in industry rather than in humans, together propose the transmission of colistin resistance in a One Health manner (4). By including humans, animals, and environmental sectors in *mcr*-carrying plasmid structure analysis, we found highly similar plasmid backbones from a silver gull, food animals, wastewater treatment plants, and hospital sewage. The nonhuman plasmids shared a wide range of genetic elements, including large antibiotic resistance regions and heavy metal-resistant operons, with our *mcr*-carrying plasmids from patients. The shared ARGs, conferring resistance to a broad spectrum of antibiotics not limited to the drugs regularly used clinically, suggest that the presence of *mcr* in our collection is not due to colistin pressure in the hospital but is more likely to be acquired from food animals and waste sources (9). The carriage of multiple large resistance regions in the *mcr*-positive IncHI2 plasmids with *tra* regions indicates that the *mcr*-carrying IncHI2 plasmids pose a greater threat to public health (3).

Our shotgun WGS study demonstrated that WGS is an effective screen for important genes, especially the silent *mcr-9.1*, *mcr-9.2*, and *mcr-10.1* variants (9). Both *mcr-9* and *mcr-10* have been reported as mediating low-level resistance to colistin, although the mechanism remains unclear (3). The activity of *mcr-10* against colistin could be even lower than that of *mcr-9*, as indicated by the colistin killing assay (2). Our susceptibility test results were similar to those of the previous study, with lower colistin MIC values for *mcr-9* and *mcr-10* (0.5 μg/mL to 1 μg/mL in K. pneumoniae, K. quasipneumoniae subsp. *quasipneumoniae*, and K. variicola versus 2 μg/mL to 4 μg/mL in Enterobacter roggenkampii and E. coli) (6). The WGS data provide all gene and promoter sequences of the colistin resistance associated genes and allow for comparative genomic analysis (Fig. 4; see Fig. S1 in the supplemental material). However, due to the lack of *mcr-10*-carrying Klebsiella genomes with colistin-resistant phenotypes, further investigation is needed to understand the key reason for the colistin susceptibility of the *mcr-10*-positive Klebsiella isolates.

The complementary long-read sequencing technology also helped to elucidate the *mcr*-carrying plasmid structures. The plasmid structures of *mcr-10.1* were divergent (less than 70% coverage) (2), which may be due to the *mcr* carriage on the hyperdivergent IncF-type plasmids (13) and in different species of Klebsiella. However, the *mcr-10.1*-adjacent MGE was conserved; an XerC-type recombinase was consistently identified directly upstream of *mcr-10.1* (6). Interestingly, only *xerC att*L was identified in our *mcr-10.1*-carrying plasmids, indicating that the acquisition of *mcr-10.1* might have occurred after the *xerC* integration of *mcr-10.1*. The identification of the perfect 9-bp IR pair further confirmed that *mcr-10.1* in our collection was acquired by IS*Ec36* insertion of the *mcr-10.1-xerC* fragment. The insertion of IS*Ec36*-*mcr-10.1*-*xerC* may also bring an additional type I restriction modification (RM) system encoded by *hsdSMR*. IS*Ec36* was initially identified in E. coli strain W635, carrying the carbapenemase gene *bla*_IMI-2_ (16).

### Conclusion.

This study characterized 20 *mcr*-carrying plasmids from a One Health perspective. This study reports the colistin-susceptible *mcr-10.1* variant in *K. quasipneumoniae* subsp*. quasipneumoniae* and *K. variicola* for the first time and shows a pair of perfect IRs next to *mcr-10.1*, illustrating IS*Ec36* mobilizing *mcr-10.1*-*xerC* and *hsdSMR* in a stepwise manner.

## MATERIALS AND METHODS

### Bacterial genomes and study design.

A total of 2,855 unique Klebsiella genomes from a surveillance study were included (17). Isolates were recovered from patient specimens at a hospital in the central part of China between 5 January 2013 and 24 July 2018. Multilocus sequence typing (MLST) was done using the Klebsiella Pasteur MLST database (https://bigsdb.pasteur.fr/klebsiella/). ARGs (including the *mcr* variants) and plasmid incompatibility groups were assigned using ResFinder version 4.0 (18) and PlasmidFinder version 2.1 (19), respectively, via the ABRicate pipeline (https://github.com/tseemann/abricate).

### Ethics.

Written informed consent was obtained from all participants. This study was approved by the ethics committees under the tracking numbers 201806861 and BGI-IRB 18061-T2.

### Scaffolding of the *mcr*-carrying plasmids.

The sizes of the *mcr*-carrying contigs ranged from 1,948 bp to 59,684 bp (20). The contigs were searched using BLAST against the nonredundant/nucleotide (nr/nt) database to find suitable plasmid references. Scaffolding of the *mcr*-carrying plasmid contigs was completed using assembly_improvement version 1.7.0 (21) and was manually checked by mapping reads to the scaffolds using Geneious 9.1.8. In addition, three genomes carrying *mcr-1.1* (strain 5-30), *mcr-9.1* (strain 36-80), and *mcr-10.1* (strain 31-62) were sequenced on a MinION device (Oxford Nanopore Technologies, UK). Unicycler version 0.4 was deployed to achieve the hybrid assembly of both the short reads (BGISEQ-500 [MGI, China]) and long reads (MinION) (https://github.com/rrwick/Unicycler). The *mcr*-carrying scaffolds were also checked and assessed by mapping the raw reads back to the assembled scaffolds.

### Antimicrobial susceptibility assays.

Antimicrobial susceptibility tests (ASTs), with the exception of colistin, were performed using the Vitek 2 Compact system (BioMérieux, Marcy-l’Etoile, France) according to the manufacturer’s instructions. The susceptibility results were interpreted according to the Clinical and Laboratory Standards Institute guidelines (22). The MIC values for colistin were determined using the broth microdilution method in cation-adjusted Mueller-Hinton broth, and the results were interpreted following the European Committee on Antimicrobial Susceptibility Testing (EUCAST) breakpoints (susceptible, ≤2 μg/mL; resistant, >2 μg/mL; https://www.eucast.org/clinical_breakpoints). No induction assays were performed, as *qseBC* was not identified in the 20 *mcr*-carrying genomes.

### Data availability.

The data that support the findings of this study have been deposited at the China National GeneBank DataBase (CNGBdb) in the CNGB Sequence Archive (CNSA) under accession number CNP0001198 (23, 24).
