# Supplementary material for: One Health Analysis of mcr-Carrying Plasmids and Emergence of mcr-10.1 in Three Species of Klebsiella Recovered from Humans in China
Source: Microbiol Spectr. 2022 Oct 26;10(6):e02306-22. doi: 10.1128/spectrum.02306-22 (PMC9769640; doi:10.1128/spectrum.02306-22)
Supplement: Supplemental file 1 — . Download spectrum.02306-22-s0001.pdf, PDF file, 1.2 MB [file spectrum.02306-22-s0001.pdf]

1
2

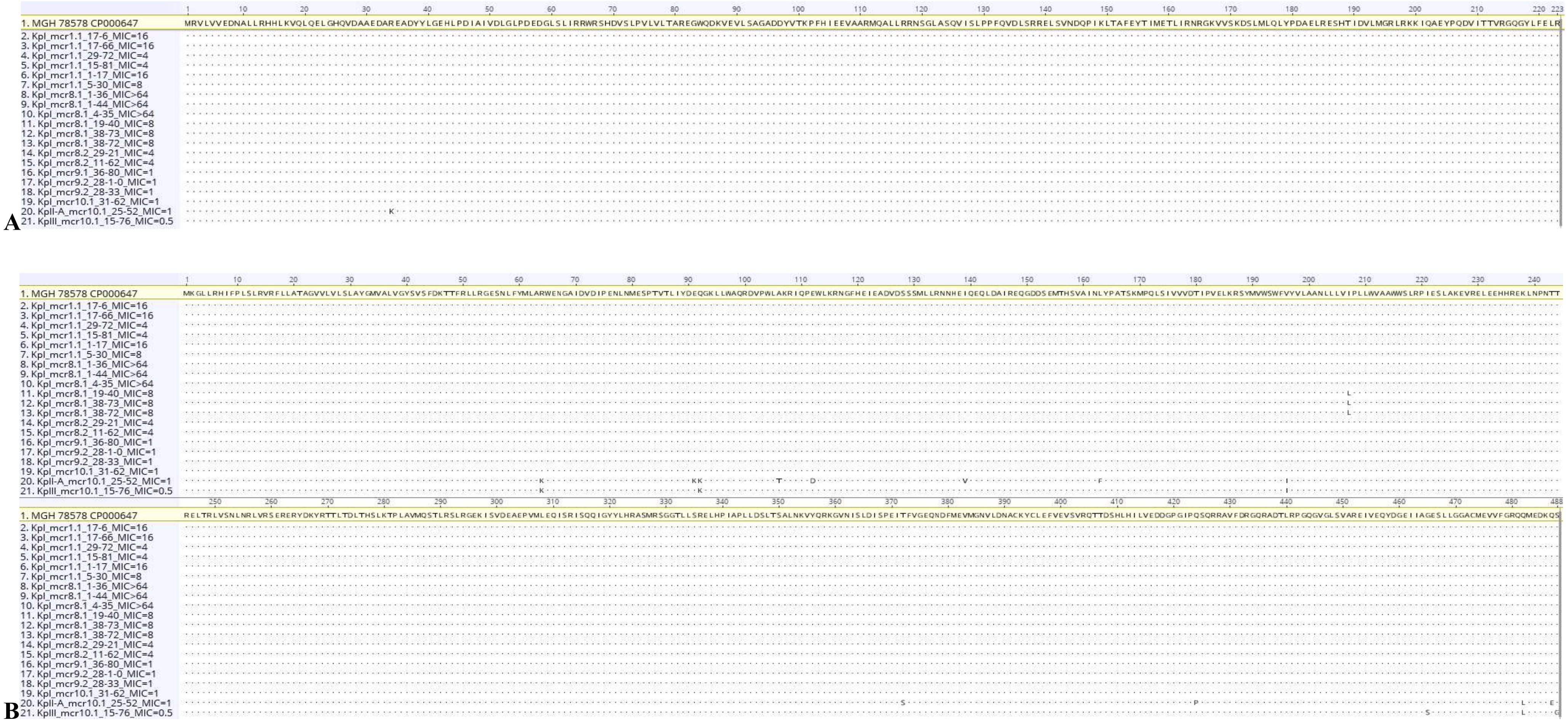
A
1. MGH 78578 CP000647
2. Kpl_mcr1.1_17-6_MIC=16
3. Kpl_mcr1.1_17-66_MIC=16
4. Kpl_mcr1.1_29-72_MIC=4
5. Kpl_mcr1.1_15-81_MIC=4
6. Kpl_mcr1.1_1-17_MIC=16
7. Kpl_mcr1.1_5-30_MIC=8
8. Kpl_mcr8.1_1-36_MIC>64
9. Kpl_mcr8.1_1-44_MIC>64
10. Kpl_mcr8.1_4-35_MIC>64
11. Kpl_mcr8.1_19-40_MIC=8
12. Kpl_mcr8.1_38-73_MIC=8
13. Kpl_mcr8.1_38-72_MIC=8
14. Kpl_mcr8.2_29-21_MIC=4
15. Kpl_mcr8.2_11-62_MIC=4
16. Kpl_mcr9.1_36-80_MIC=1
17. Kpl_mcr9.2_28-1-0_MIC=1
18. Kpl_mcr9.2_28-33_MIC=1
19. Kpl_mcr10.1_31-62_MIC=1
20. KpII-A_mcr10.1_25-52_MIC=1
21. KpIII_mcr10.1_15-76_MIC=0.5
B

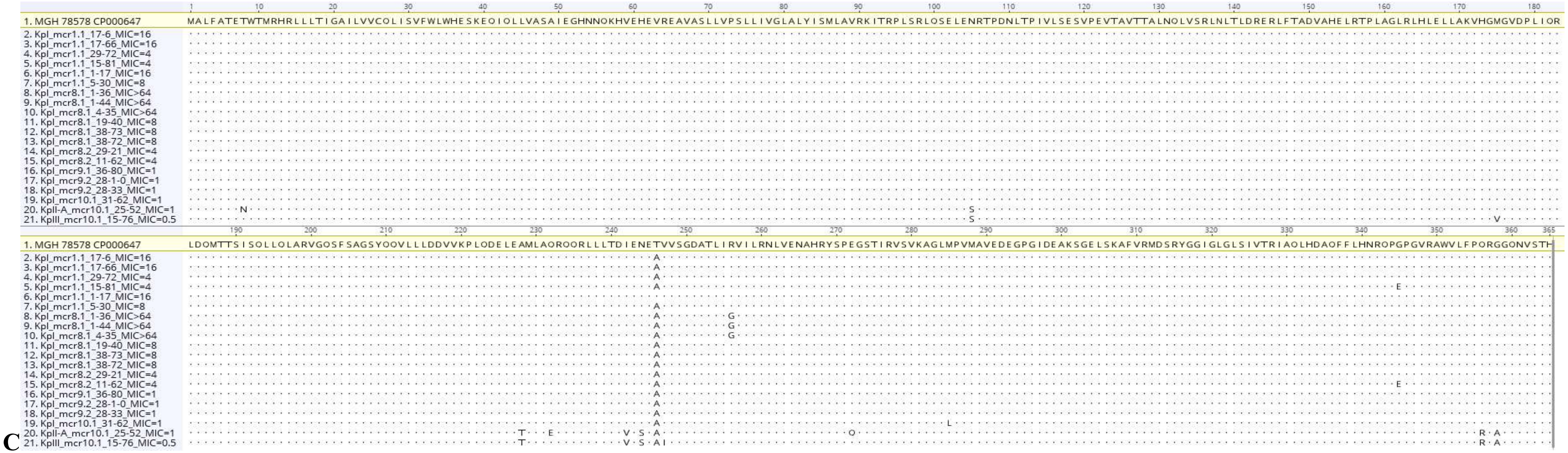

C

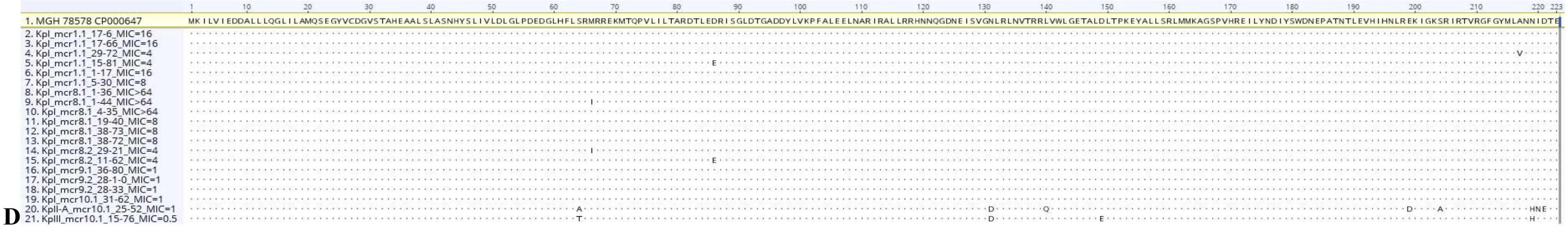

D

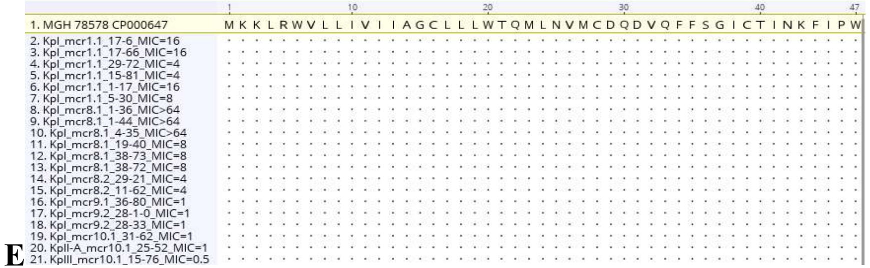

E

Supplementary Figure 1 Amino acid sequence alignments of *K. pneumoniae* chromosomal genes associated with colistin susceptibility

The reference gene sequences are from strain MGH 78578 (GenBank accession number CP000647.1). The species, *mcr* variants, strain names and the colistin

MIC values of the *mcr*-carrying isolates in this study are labeled. Identical bases are indicated by dots. (A) *phoP* (B) *phoQ* (C) *pmrA* (D) *pmrB* (E) *mgrB*

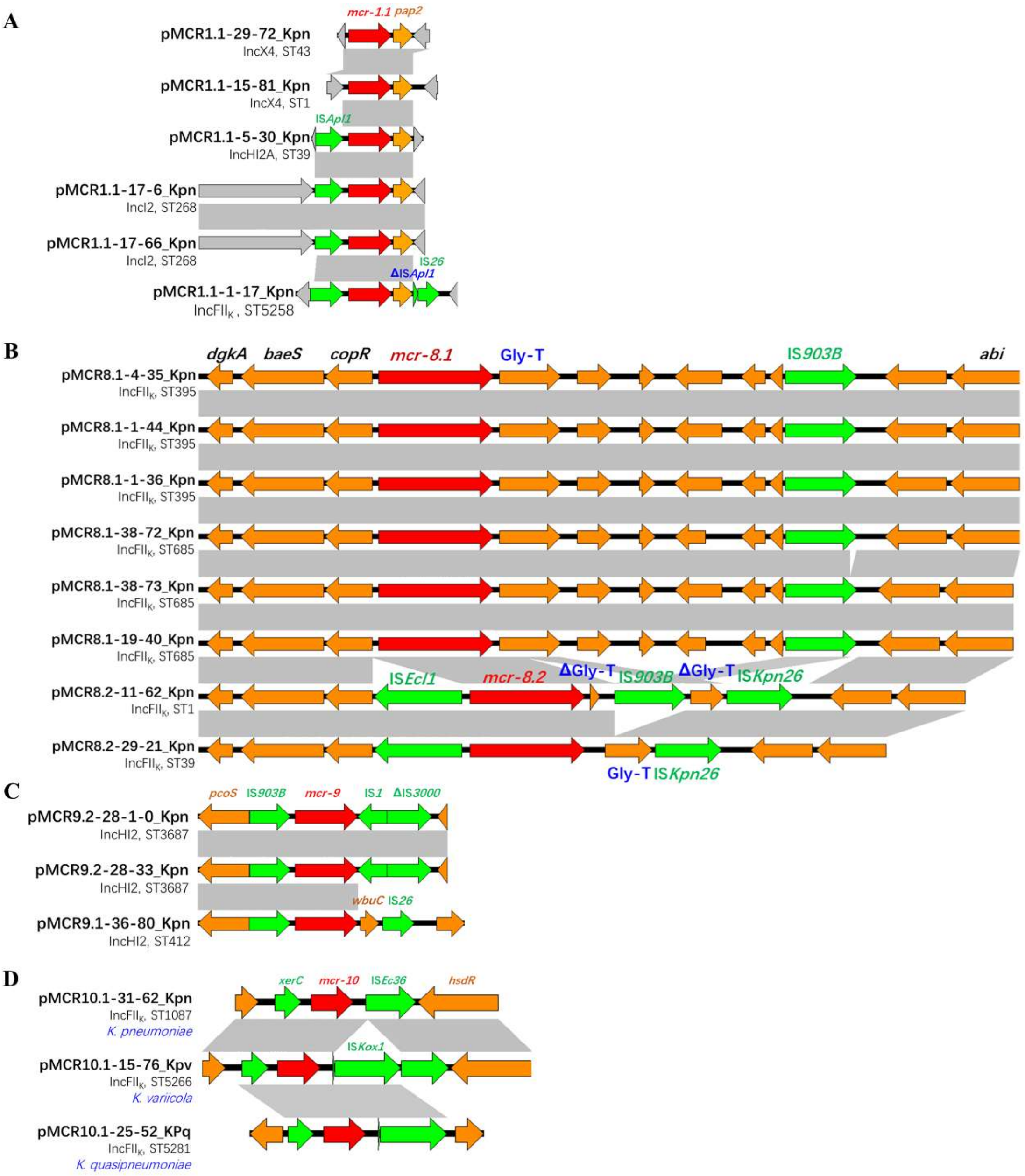

Supplementary Figure 2 Characterization of *mcr* genetic contexts in this study

The plasmid name and incompatibility groups are labeled for each sequence. The genes and gentic elements are colored as follows: red for *mcr*, green for the mobile genetic elements (MGEs), orange for the *mcr* adjacent genes, and gary for the hypothetical proteins. Grey shadings connect highly similar sequences. (A) *mcr-1* (B) *mcr-8* (C) *mcr-9* (D) *mcr-10*
